# Primary Leiomyosarcoma of the Adrenal Gland

**DOI:** 10.1155/S1357714X01000184

**Published:** 2001-06

**Authors:** Boudewijn van Etten, Marc G. A. van Ijken, Wolter J. Mooi, Matthijs Oudkerk, Albertus N. van Geel

**Affiliations:** 1 Department of Surgical Oncology Rotterdam The Netherlands; 2 Department of Surgical Radiology Rotterdam The Netherlands; 3 Department of Pathology The Netherlands Cancer Institute Amsterdam The Netherlands; 4 Department of Surgical Oncology University Hospital Rotterdam–Daniel den Hoed Cancer Center PO Box 5201 Rotterdam 3008 AE The Netherlands

## Abstract

We report a rare case of a primary leiomyosarcoma of the adrenal gland. A 73-year-old woman presented with an inferior vena cava syndrome. MR imaging was suggestive of a large tumour originating from the right adrenal gland. Angiography revealed a tumour vascularised by the right adrenal artery. At explorative laparotomy a tumour of 27 cm in diameter was found which was completely fixed to the liver; the tumour was therefore considered unresectable. As a consequence of the mechanical problems caused by this large tumour, the patient died 3 weeks after the operation. Autopsy revealed no distant metastases or other primary tumour site.


*Sarcoma* (2001) 5, 95–99

CASE REPORT

### Primary leiomyosarcoma of the adrenal gland

BOUDEWIJN VAN ETTEN[1], MARC G.A. VAN IJKEN[1], WOLTER J. MOOI[3], MATTHIJS OUDKERK[2] & ALBERTUS N. VAN GEEL[1]

*Departments of Surgical [1]Oncology and [2]Radiology, Rotterdam, The Netherlands, and [3]Department of Pathology, The Netherlands Cancer Institute, Amsterdam, The Netherlands*

**Abstract**
We report a rare case of a primary leiomyosarcoma of the adrenal gland. A 73-year-old woman presented with an inferior vena cava syndrome. MR imaging was suggestive of a large tumour originating from the right adrenal gland. Angiography revealed a tumour vascularised by the right adrenal artery. At explorative laparotomy a tumour of 27 cm in diameter was found which was completely fixed to the liver; the tumour was therefore considered unresectable. As a consequence of the mechanical problems caused by this large tumour, the patient died 3 weeks after the operation. Autopsy revealed no distant metastases or other primary tumour site.

**Key words:** *leiomyosarcoma, adrenal, angiography*

### Introduction

Primary mesenchymal neoplasms of the adrenal gland constitute a heterogeneous group of rare entities, including myelolipoma, haemangioma, lymphangioma, angiosarcoma, neurilemoma, leiomyoma and leiomyosarcoma.[1] A primary adrenal leiomyosarcoma is exceptionally rare, with only four documented cases available in the literature.[2–5] Here we describe a case, that presented with an inferior vena cava syndrome. Based on detailed angiography of the arterial supply of the tumour, as well as the autopsy data, the tumour was classified as originating from the right adrenal gland. The histological features showed characteristics of leiomyosarcoma.

### Case report

A 73-year-old woman was referred to our hospital for further evaluation and assessment of surgical therapeutic options of an intra- or retroperitoneal soft tissue tumour, which caused inferior vena cava syndrome. Three months prior to admission, the patient had noticed swelling of both legs, and she experienced slight distension of her abdomen as well as bouts of abdominal pain. In addition to this, she lost her appetite and suffered from fatigue. There had been no significant weight loss. Her medical history included an abdominal gunshot trauma sustained 4 years previously, which had resulted in laceration of the right liver lobe. The liver had been partially resected and a cholecystectomy was performed. There was no history of excessive alcohol intake or hepatitis.

At physical examination, we saw a pale woman with blood pressure of 110/65 mmHg. The abdomen was distended, with visible enlarged abdominal wall veins. A large firm mass was palpated in the right upper abdomen. There was pitting oedema and distension of the veins of both legs, consistent with an inferior vena cava syndrome.

Laboratory studies showed an erythrocyte sedimentation rate of 47 mm/h (10–20 mm/h), a leucocytosis of $22.4\times10^9$/l ($4–10\times10^9$/l), a haemoglobin concentration of 6.6 mmol/l (7.3–9.3 mmol/l), an alkaline phosphatase of 193 U/l (25–75 U/l), an alaline aminotransferase of 33 U/l (5–30 U/l), an aspartate aminotransferase of 58 U/l (5–30 U/l), a $\gamma$-glutamyl transpeptidase of 79 U/l (5–35 U/l), a lactate dehydrogenase of 730 U/l (160–320 U/l), a total bilirubin of 22 μmol/l (4–14 μmol/l), a creatinine of 155 μmol/l (60–110 μmol/l) and a urea concentration of 2.3 mmol/l (2.5–8 μmol/l). The urine sediment was normal. Computed tomography (CT) revealed, on transversal images, a large mass located in the

Correspondence to: A.N. van Geel, M.D., Ph.D., Department of Surgical Oncology, University Hospital Rotterdam–Daniel den Hoed Cancer Center, PO Box 5201, 3008 AE Rotterdam, The Netherlands. Tel.: + 31-10-439-1911; Fax: +31-10-439-1011; E-mail: geel@chih.azr.nl

1357–714X print/1369–1643 online/01/020095–05 © 2001 Taylor & Francis Ltd
DOI: 10.1080/13577140120048601

right abdomen, possibly originating from the right kidney. An inferior vena cavogram showed complete occlusion of the inferior vena cava. Placing of a stent in the vena cava was technically not feasible. Magnetic resonance (MR) images were suggestive of an adrenal rather than a renal tumour. On coronal images a large tumour of about 25 cm in longitudinal axis was found (Fig. 1). These findings corresponded with the angiogram which revealed that the arterial blood supply of the tumour was derived from the right adrenal artery (Fig. 2). This arterial vascularisation produced evidence of the adrenal origin of the tumour.

A percutaneous thick-needle biopsy of the tumour had been performed in the referring hospital, and revealed a spindle cell tumour with features of smooth muscle differentiation; leiomyoma and leiomyosarcoma were considered in the differential diagnosis. CT and MRI showed no evidence of metastases to lungs, brain or lymph nodes.

An exploratory laparotomy was performed. A subhepatic tumour was found to extend from the midline to the right abdominal wall, with complete fixation to the liver. Because of this, resection was considered impossible, also in view of the poor clinical condition of the patient. Palliative and supportive care was instituted; the patient died 3 weeks after the laparotomy.

Autopsy revealed a predominantly sub-hepatic grey–white tumour with a maximal diameter of 27 cm. The right adrenal gland could not be identified. The left adrenal gland appeared to be normal. No tumour mass was found in the uterus or bowel or any other site at autopsy. Histological examination again revealed a spindle cell tumour with a moderate degree of atypia and with up to 10 mitoses per 2 $mm^2$, with areas of coagulation necrosis (Fig. 3a).

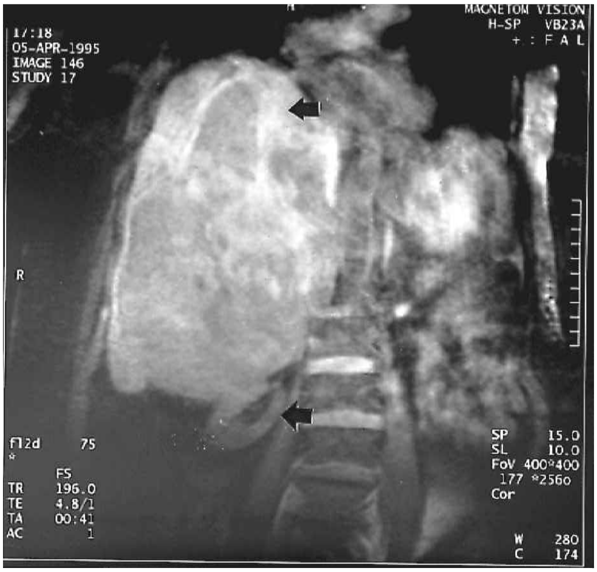

**Fig. 1.** *T2-weighted coronal MR image of the upper abdomen displaying a large tumour which compresses the normal liverparenchyma (upper ➜) and the right kidney (lower ➜).*

Tumour cells were elongated, had blunt-ended nuclei and the cytoplasm was eosinophilic with a slightly fibrillary appearance. Immunohistochemistry showed strong immunoreactivity for smooth muscle actin (Fig. 3b), attesting to the smooth muscle differentiation of the tumour. At autopsy, metastases or other primary sites were not identified. There was a right-sided pleural effusion resulting in atelectasis of the subjacent right lung. The vascular wall of the inferior vena cava was intact, excluding a possible primary tumour of the inferior vena cava. Thrombosis of the inferior caval vein was found, which could be explained by extrinsic compression by the tumour.

### Discussion

Primary mesenchymal tumours of the adrenal gland are very rare. Most are benign, with myelolipomas and haemangiomas being the most common entities in this group.[6] A literature search identified four previous reports of primary adrenal leiomyosarcoma. These occurred in three males and one female, ranging in age from 30 to 49 years. In about 200 cases described in literature a leiomyosarcoma originated from the inferior vena cava. These patients often presented with a inferior vena cava syndrome as well.[7] Other primary sites of vascular leiomyosarcoma are even more rare.[8]

In general, adrenal masses larger than 3 cm are suspicious of malignancy.[9,10] To evaluate the resectability of a suspicious adrenal tumour, pre-operative imaging of the tumour and screening for distant metastases is essential. In view of the rarity of primary adrenal leiomyosarcoma, it is obvious that the possibility of a clinically occult tumour elsewhere needs to be carefully ruled out before the this diagnosis is made.[11] In our case CT and MRI could not detect any other sites of tumour growth. CT alone cannot provide definitive proof that a large upper abdominal tumour is of adrenal origin and angiography is of great value in the pre-operative work-up of such patients.[12] But one must bear in mind that a metastasis in the adrenal gland would also be supplied by the adrenal artery. The combination of CT, MRI and angiography proved useful in this patient.

In our case, there is a previous history of a gunshot trauma necessitating a partial hepatectomy of the right liver lobe. In the literature there is a scattering of case reports that suggest that occasionally, severe tissue trauma such as may result from gunshots could be a causal or contributing factor in the pathogenesis of some sarcomas.[13–21] At time of the emergency laparotomy for the lacerated liver, there had been no evidence of a tumour. Whether the adrenal gland had been damaged by the gunshot wound is not clear from the records. In our case the latency period between trauma and diagnosis of malignancy was 4 years; most cases of sarcoma arising after tissue trauma report a much longer period.

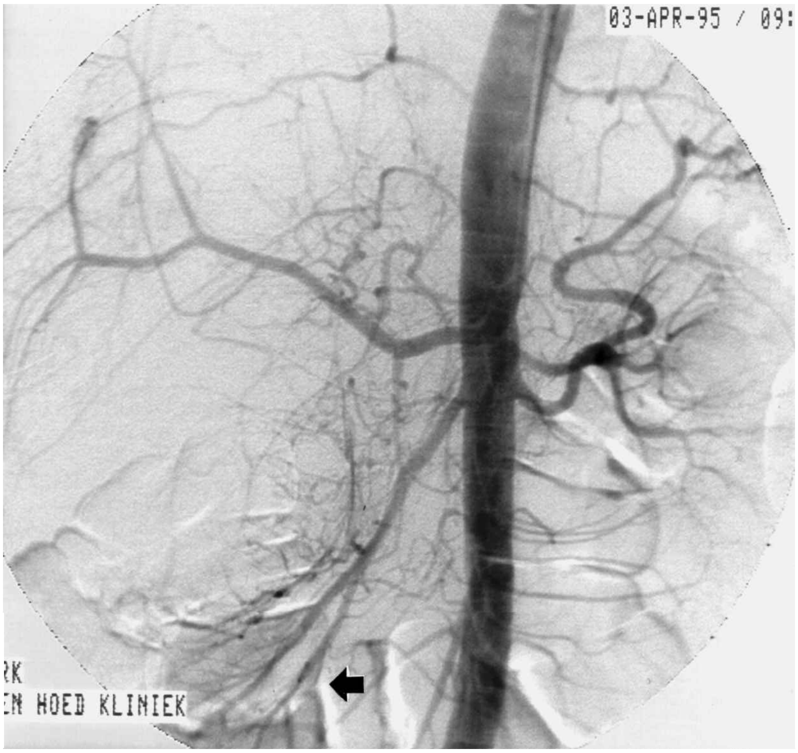

A

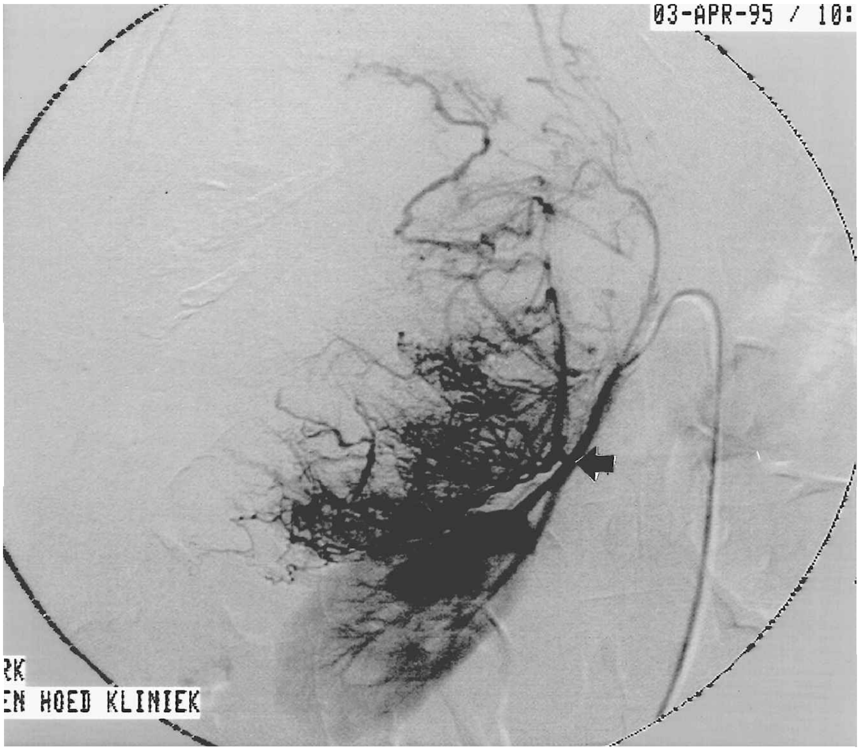

B

**Fig. 2.** *(A) Angiography of the abdominal aorta demonstrating a tumour in the right upper abdomen compressing the right kidney (➔). (B) Selective angiography of the right renal artery demonstrates vascularisation of the tumour by the right adrenal artery (➔).*

Histopathological evaluation is indispensable not only for determining tumour type but also for grading and, in parallel biological aggressive behaviour. In this respect the mitotic index and the presence of necrosis are important parameters. Lack *et al.* described a case of a high grade adrenal leiomyosarcoma with distant metastases at time of diagnosis which could be treated only palliatively.[4] One of the other previously reported

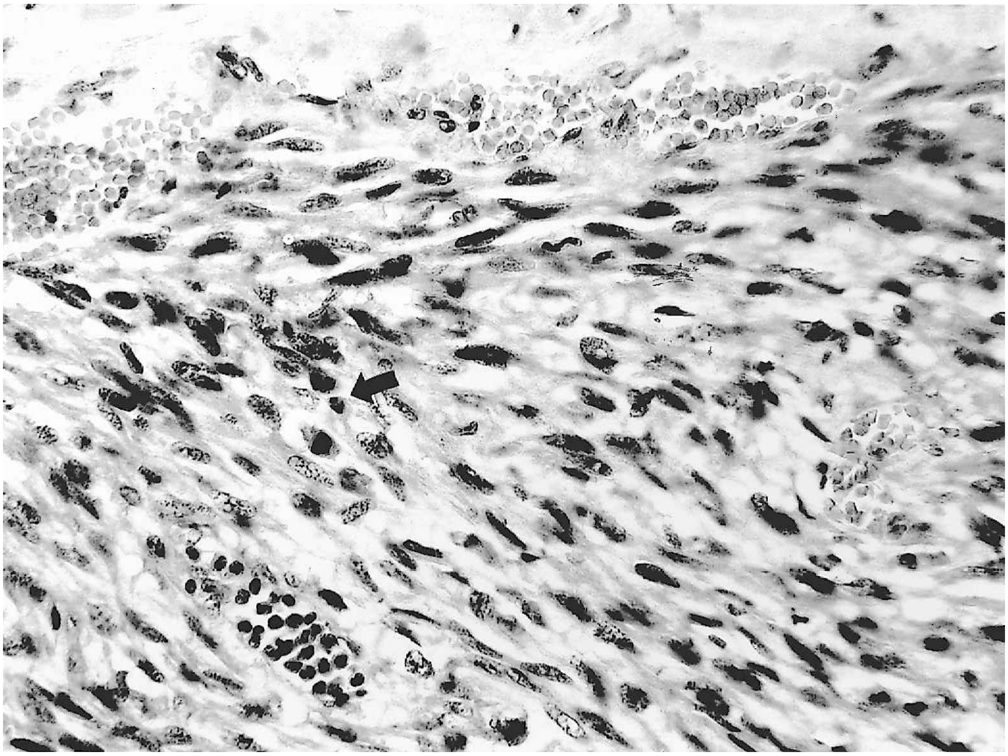

A

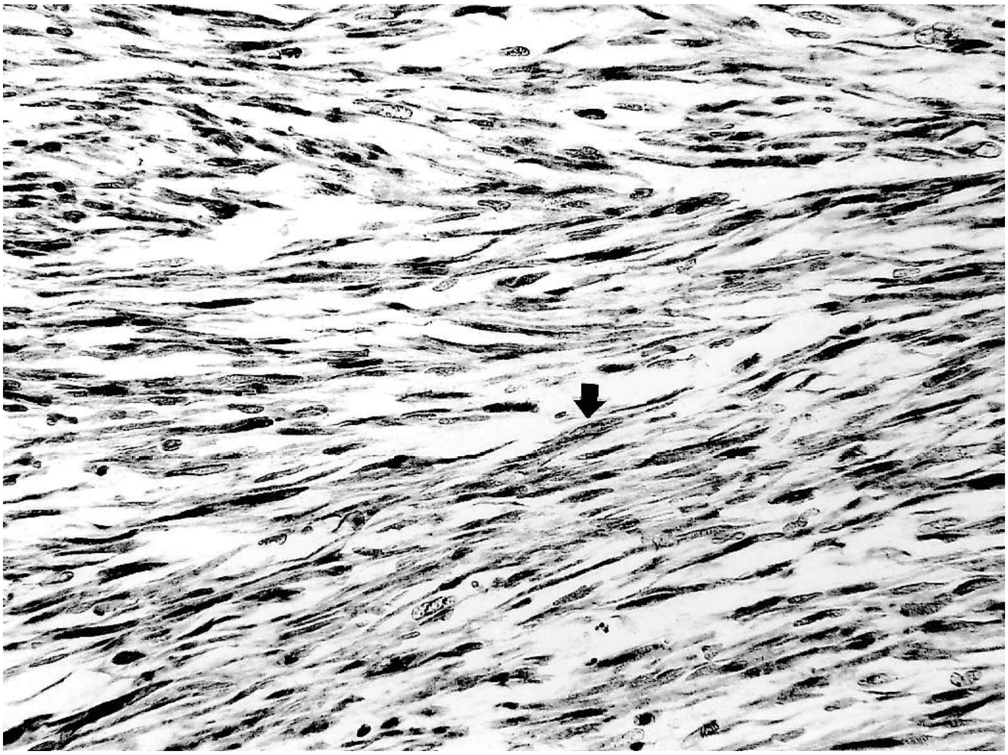

B

**Fig. 3.** *(A) Histological appearance of the tumour. A proliferation of elongated tumour cells with predominantly blunt-ended nuclei is seen. A mitosis is present left of centre (➜). Haematoxylin and eosin, magnification, × 700. (B) The tumour cells are strongly positive (➜) for smooth muscle actin, a protein involved in smooth muscle cell contraction and a histopathological marker of smooth muscle cell differentiation. Avidin–biotin peroxidase immunostaining, magnification, × 450.*

cases of adrenal leiomyosarcoma concerned a low-grade tumour that showed no evidence of recurrence at 20 months after operation.[5] In this fifth reported case of a primary leiomyosarcoma of the adrenal gland the patient did not die because of malignant behaviour of the tumour but because of the mechanical problems caused by this very large tumour. The patient suffered from a vena cava inferior syndrome, atelectasis of the right lung with 2.5-l pleural fluid and bronchopneumonia of the left lung.

It seems that aggressive local surgical management of low-grade sarcoma can produce prolonged survival in these patients. Because a radical resection or compartimental resection with wide tumour-free margins in the retroperitoneal space is not possible for large tumours, the local recurrence is high. From soft tissue sarcomas of the extremities it is known that adjuvant radiotherapy after a marginal resection results in similar survival rates as after a radical resection only.[22] Therefore radiotherapy should always be considered after a marginal resection of retroperitoneal soft tissue sarcoma. The role of adjuvant chemotherapy in soft tissue sarcoma still remains unclear. A meta-analysis of 14 randomised studies suggested a possible survival improvement, however not significant.[23]

### References

1 Lack EE. *Tumors of adrenal gland and extra-adrenal paraganglia*. Washington: Armed Forces Institute of Pathology, 1995.
2 Choi SH, Liu K. Leiomyosarcoma of the adrenal gland and its angiographic features: a case report. *J Surg Oncol* 1981; 16:145–8.
3 Fernández JM, Huescar AM, Ablanedo P, Rábade CJ, Pérez García FJ, Rodríguez Martínez JJ, *et al.* Primary leiomyosarcoma. A rare tumor of the adrenal gland. *Arch Esp Urol* 1998; 51:1029–31.
4 Lack EE, Graham CW, Azumi N, Bitterman P, Rusnock EJ, O'Brien W, *et al.* Primary leiomyosarcoma of adrenal gland. Case report with immunohistochemical and ultrastructural study. *Am J Surg Pathol* 1991; 15:899–905.
5 Zetler PJ, Filipenko JD, Bilbey JH, Schmidt N. Primary adrenal leiomyosarcoma in a man with acquired immunodeficiency syndrome (AIDS). Further evidence for an increase in smooth muscle tumors related to Epstein-Barr infection in AIDS [see comments]. *Arch Pathol Lab Med* 1995; 119:1164–7.
6 Goren E, Bensal D, Reif RM, Eidelman A. Cavernous hemangioma of the adrenal gland. *J Urol* 1986; 135:341–2.
7 Hines OJ, Nelson S, Quinones-Baldrich WJ, *et al.* Leiomyosarcoma of the inferior vena cava: prognosis and comparison with leiomyosarcoma of other anatomic sites. *Cancer* 1999; 85:1077–83.
8 van Gulik TM, Taat CW, Slors JF, *et al.* Leiomyosarcoma of large and small veins: clinical findings and results of treatment in six patients. *Eur J Surg Oncol* 1991; 17:125–34
9 Singer AA, Obuchowski NA, Einstein DM, Paushter DM. Metastasis or adenoma? Computed tomographic evaluation of the adrenal mass. *Clevel Clin J Med* 1994; 61:200–5.
10 Yamakita N, Saitoh M, Mercado-Asis LB, Kitada M, Morita H, Yasuda K, *et al.* Asymptomatic adrenal tumor; 386 cases in Japan including our 7 cases. *Endocrinol Jpn* 1990; 37:671–84.
11 Rao NG, Krishnaswami S, Cherian G, Krishnaswami H. Sarcoma of the pulmonary artery with metastases to pancreas and adrenal glands. *Chest* 1974; 66:452–62.
12 Kolmannskog F, Kolbenstvedt A, Brekke IB. CT and angiography in adrenocortical carcinoma. *Acta Radiol* 1992; 33:45–9.
13 Gurin IL. Osteochondroplastic sarcoma of the skin, developing in an area of chronic ulcer following gunshot wound. *Ark Patol* 1968; 30:73–5.
14 Dijkstra MD, Balm AJ, Gregor RT, Hilgers FJ, Loftus BM. Soft tissue sarcomas of the head and neck associated with surgical trauma. *J Laryng Otol* 1995; 109:126–9.
15 Dreyfuss UY, Auslander L, Bialik V, Fishman J. Ewing's sarcoma of the hand following recurrent trauma; a case report. *Hand* 1980; 12:300–3.
16 Hamblen DL, Carter RL. Sarcoma and joint replacement. *J Bone Joint Surg (Br vol)* 1984; 66:625–7.
17 Rill A. Testicular sarcoma as the consequence of a gunshot wound. *Lijecnicki Vjesnik* 1970; 92:479–82.
18 Jennings TA, Peterson L, Axiotis CA, Friedlaender GE, Cooke RA, Rosai J. Angiosarcoma associated with foreign body material. A report of three cases. *Cancer* 1988; 62:2436–44.
19 Erle A, Buchner HJ. Sarcoma following injury (case report). *Stomatol DDR* 1978; 28:898–9.
20 Ohkuda K, Watanabe S, Saitoh Y, Suda S, Satoh H, Nitta S, *et al.* Possible development of pulmonary scar cancer in a patient with pulmonary bullet wound. *Nihon Kyobu Shikkan Gakkai Zasshi* 1980; 18:347–51.
21 Behnke KD, Schneider H. Fistula cancer—a rare complication of gunshot fracture osteomyelitis. *Beitr Orthop Traumatol* 1985; 32:469–71.
22 Sodaski C, Suit HD, Rosenberg A, *et al.* Pre-operative radiation, surgical margins, and local control of extremity sarcomas of soft tissue sarcomas. *J Surg Oncol* 1993; 52:223–30.
23 Verweij J, Seynaeve C. The reason for confining the use of adjuvant chemotherapy in soft tissue sarcoma to the investigational setting. *Semin Radiat Oncol* 1999; 9:352–9.
